# Pollen Coat Proteomes of *Arabidopsis thaliana*, *Arabidopsis lyrata*, and *Brassica oleracea* Reveal Remarkable Diversity of Small Cysteine-Rich Proteins at the Pollen-Stigma Interface

**DOI:** 10.3390/biom13010157

**Published:** 2023-01-12

**Authors:** Ludi Wang, Yui-Leung Lau, Lian Fan, Maurice Bosch, James Doughty

**Affiliations:** 1 Institute of Biological, Environmental and Rural Sciences (IBERS), Aberystwyth University, Plas Gogerddan, Aberystwyth SY23 3EE, UK; 2 Department of Life Sciences, University of Bath, Claverton Down, Bath BA2 7AY, UK

**Keywords:** adaptive evolution, *Arabidopsis*, *Brassica*, cell wall, cysteine-rich proteins, lipid metabolism, pollen coat proteomes, pollen-stigma interaction, reproduction, signalling

## Abstract

The pollen coat is the outermost domain of the pollen grain and is largely derived from the anther tapetum, which is a secretory tissue that degenerates late in pollen development. By being localised at the interface of the pollen–stigma interaction, the pollen coat plays a central role in mediating early pollination events, including molecular recognition. Amongst species of the Brassicaceae, a growing body of data has revealed that the pollen coat carries a range of proteins, with a number of small cysteine-rich proteins (CRPs) being identified as important regulators of the pollen–stigma interaction. By utilising a state-of-the-art liquid chromatography/tandem mass spectrometry (LC-MS/MS) approach, rich pollen coat proteomic profiles were obtained for *Arabidopsis thaliana*, *Arabidopsis lyrata*, and *Brassica oleracea*, which greatly extended previous datasets. All three proteomes revealed a strikingly large number of small CRPs that were not previously reported as pollen coat components. The profiling also uncovered a wide range of other protein families, many of which were enriched in the pollen coat proteomes and had functions associated with signal transduction, cell walls, lipid metabolism and defence. These proteomes provide an excellent source of molecular targets for future investigations into the pollen–stigma interaction and its potential evolutionary links to plant–pathogen interactions.

## 1. Introduction

Pollination during sexual plant reproduction involves complex interactions between the male gametophyte and female reproductive tissues. The compatibility of the pollen and pistil is highly regulated by both male and female structure-derived factors. Many angiosperms produce pollen grains that possess a pollen coat (also called a pollenkitt or tryphine) that constitutes the outermost layer of pollen [1]. The pollen coat is made up of a complex mixture of lipids, proteins, glycoconjugates, and pigments that has a range of functions, including protection of the pollen grain and the facilitation of pollination [2,3,4,5]. The pollen coat largely originates from a layer of highly secretory tapetal cells that line the anther locule [3,6], though it is now recognised that the pollen grain itself also secretes proteins that localise to the coat in mature pollen [7,8,9]. In members of Brassicaceae with ‘dry’ type stigmas, the pollen coat not only facilitates the adhesion of pollen grains to the stigma, but it also delivers factors that act in the earliest phase of the pollen–stigma recognition [10,11,12]. The dry stigma provides a highly discriminatory environment that can prevent the hydration and potential pollen tube growth of heterospecific or intraspecific incompatible pollen. Following contact between the pollen grain and the stigma papilla cell, a molecular dialogue is rapidly established that culminates in either the acceptance of compatible pollen grains or the rejection of incompatible ones [12,13,14,15].

Amongst members of the Brassicaceae, multiple pollen-borne factors that influence the pollen–stigma interaction have been identified through analyses of pollen coat components and via mutational studies. These factors can be broadly divided into two functional groups: one that influences the biophysical properties of the pollen coat, and the other that contains likely signalling components. For instance, mutants that impact the production of very long chain lipids in the pollen coat have severely impaired pollen hydration, either by affecting the solubility of other factors necessary for the pollen–stigma interaction, or by disrupting hydration conduits within the coating [16,17,18]. In addition, proline-rich extracellular lipases (EXLs), in conjunction with an oleosin-domain-containing glycine-rich protein (GRP17), appear to work cooperatively to modify the properties of the pollen coat to facilitate the transport of water from the stigmatic papillae [16,19,20,21]. Those factors identified as having a signalling role all strikingly belong to a diverse grouping of small cysteine-rich proteins (CRPs). Several pollen coat CRPs, including the *S*-locus protein 11/*S*-locus cysteine-rich proteins (*SP11/SCR*), pollen coat protein class As (PCP-As), and PCP-Bs, have been found to regulate very early pollination events in *Brassica* and *Arabidopsis* species by mediating cell–cell communication between the pollen grain and stigma [7,8,9,22,23,24,25]. Pollen coat CRPs that are well-characterised have been shown to act as ligands to transmembrane receptors located at the plasma membrane of stigmatic papilla cells. Subsequent downstream signalling leads to either activation of a basal compatibility pathway, involving the release of water to the pollen, or, in species with self-incompatibility (SI), the rejection of incompatible pollen via pathways that involve phosphorylation, the modulation of stigmatic ROS levels, and targeted protein degradation (reviewed in [12,15]). Importantly, small CRPs are emerging as key regulators involved in multiple stages of plant reproduction beyond the initial pollen–stigma interaction (reviewed in [26,27,28]). Despite these insights, the pollen coat contains many unidentified/uncharacterised proteins that likely function in early pollen–pistil interactions, and, thus, obtaining a more complete pollen coat proteome is a priority.

Although the importance of pollen coat proteins in plant reproduction is now well established, very few pollen coat proteomic profiles are available to date. Moreover, the coverage of small proteins and peptides in previous studies, especially with a size below 10 kDa, is generally poor. The proteomic analysis of mature pollen grains of *Oryza sativa* and *Arabidopsis thaliana*, though not focused on the isolated pollen coat, may contain some pollen coat components [29,30]. More specific pollen coat profiling was carried out in *Brassica napus* [31], *Arabidopsis thaliana* [32], *Zea mays* [33], and *Olea europaea* [5], but less than 20 proteins were detected from each study. This may have resulted from the scarcity of pollen coat material, as well as the low sensitivity and resolution of the analytic system applied.

In recent years, proteomic analytic techniques have rapidly developed and delivered improvements in sensitivity and cost-effectiveness. In this study, a powerful liquid chromatography/tandem mass spectrometry (LC-MS/MS) system enabled us to achieve a new level of data richness and sensitivity compared to previous pollen coat proteomic analyses. To elucidate a wide range of pollen coat proteins that are commonly present in Brassicaceae, we targeted three members of the Brassicaceae (*Brassica oleracea*, *Arabidopsis lyrata*, and *Arabidopsis thaliana*), which is a major and economically important family that is commonly used in the study of pollen–stigma compatibility. These species are of interest because of their different breeding systems (*B. oleracea* and *A. lyrata* possess an SI system, whereas *A. thaliana* is self-compatible) and their evolutionary distance, with *Brassica* lineages estimated to have diverged from *Arabidopsis* approximately 23 MYA [34]. The subsequent divergence of *A. thaliana* from the clade containing *A. lyrata* occurred around 6 MYA and was followed by the loss of SI in *A. thaliana* [34,35]. In addition to larger proteins with functions associated with signalling, lipid metabolism, cell walls, and defence, proteomic profiling of the pollen coat from these three species revealed a strikingly large number of small CRPs that have not previously been reported as pollen coat components. A molecular evolutionary analysis provides evidence of positive selection in some of the genes encoding pollen coat CRPs that are shared across the three species. The richness of these datasets demonstrates the complexity of the pollen coat amongst members of the Brassicaceae, provides a resource for studies on the evolution of breeding systems, and yields a source of targets that can be explored for functional studies in plant reproduction.

## 2. Materials and Methods

### 2.1. Plant Material and Growth Conditions

*Arabidopsis thaliana* (Col-0), *Arabidopsis lyrata* (ssp. *lyrata*, Al_RON27, sampled from a population of plants at Rondeau Provincial Park, Lake Erie), and *Brassica oleracea* (var. *alboglabra* L., S29, Horticultural Research International, Wellesbourne, UK) plants were used for the isolation of pollen coat proteins. All plants were grown in a glasshouse with 16 h illumination, a day temperature of 21 °C, and a night temperature of 17 °C.

### 2.2. Extractions of Pollen Coat Proteins

*B. oleracea* pollen was collected as described by Stephenson et al. [36]. *A. lyrata* pollen was scraped from freshly dehisced anthers with a fine needle and checked under a microscope to ensure no contamination by other tissues. *A. thaliana* pollen was collected daily by a vacuum cleaner with a filter system constructed from plumbing parts with two layers of interspersed mesh filters as described by Johnson–Brousseau and McCormick [37]. Unwanted plant tissue was filtered by the 150-micron mesh filter, while pollen grains were accumulated on the 10-micron mesh filter and collected with a scalpel. The collected pollen was weighed and stored at −80 °C. The hydrophobic pollen coat was isolated using cyclohexane, which was an approach initially applied for *Brassica* [38,39] and successfully adapted for *Arabidopsis*. To isolate the pollen coat from *A. thaliana* pollen grains, 800 μL of cyclohexane was added to 80 mg of pollen. The mixture was briefly vortexed and placed in a filter unit (a holed 0.5 mL microfuge tube plugged with a small piece of glass fibre paper inserted in a 1.5 mL microfuge tube) and centrifuged at 16,000 × *g* for 14 s to elute the liquid phase. The cyclohexane was removed by freeze drying. To isolate the pollen coat from *A. lyrata* and *B. oleracea* pollen grains, the eluate was left to dry on a glass slide, and the residue was collected using scalpels. A total of 100 μL of PBS buffer (phosphate-buffered saline, Oxoid, Hampshire, UK) was added to the pollen coat sample, and the mix was sonicated on ice to achieve a milky homogenous suspension. The suspension was centrifuged until a lipid-like phase was observed on top of the aqueous pollen coat protein-containing phase that was collected as the protein extract. This centrifugation step of the aqueous phase was repeated until the product was clear, and no lipid-like phase could be observed. Protease inhibitors (cOmplete^TM^ Protease Inhibitor Cocktail Tablets, Roche Diagnostics GmbH, Mannheim, Germany) were added to the sample, which was stored at −80 °C prior to analyses. Proteins extracts were verified by gel electrophoresis and visualised using a SilverXpress^TM^ Silver Staining Kit (Life Technologies, Carlsbad, CA, USA). Three independent replicates of pollen coat protein extractions for each species were prepared for further profiling.

### 2.3. Identification of Pollen Coat Proteins by Tandem Mass Spectrometry

The pollen coat protein extracts were reduced by ‘in-solution digestion’ using 10 mM of Tris(2-carboxyethyl)phosphine hydrochloride (TCEP) that was incubated for one hour at 55 °C, followed by alkylation using 40 mM of iodoacetamide that was incubated for one hour at room temperature. A treatment with 2.5% (*w*/*w*) trypsin was carried out overnight at 37 °C. The digested samples were resuspended in 5% formic acid and desalted using Sep-Pak^®^ cartridges (Waters, Milford, MA, USA). The eluate from the Sep-Pak^®^ cartridge was evaporated, and pellets were resuspended in 1% formic acid before LC-MS/MS. Samples were fractionated by using an Ultimate^TM^ 3000 Nano-HPLC system in line with a linear trap quadrupole (LTQ)-orbitrap velos mass spectrometer (Thermo Fisher Scientific, Loughborough, UK). Peptides in 1% (*v/v*) formic acid were injected into an Acclaim^TM^ PepMap^TM^ C18 nano-trap column (Thermo Fisher Scientific, Loughborough, UK). After washing with 0.5% (*v/v*) acetonitrile, 0.1% (*v/v*) formic acid peptides were resolved on an Acclaim^TM^ PepMap^TM^ C18 reverse phase analytical column (250 mm × 75 μm, Thermo Fisher Scientific, Loughborough, UK) over a 150 min organic gradient by mixing solvent A (0.1% formic acid) and solvent B (aqueous 80% acetonitrile in 0.1% formic acid). Seven gradient segments were used (1–6% solvent B over one min, 6–15% B over 58 min, 15–32% B over 58 min, 32–40% B over 5 min, 40–90% B over 1 min, held at 90% B for six min, and then reduced to 1% B over one min.) with a flow rate of 300 μL min^−1^. Peptides were ionised by nano-electrospray ionization at 2.1 kV using a stainless-steel emitter with an internal diameter of 30 μm (Thermo Fisher Scientific, Loughborough, UK) at a capillary temperature of 250 °C. Tandem mass spectra were acquired using the LTQ-orbitrap velos mass spectrometer controlled by Xcalibur^TM^ 2.1 software and operated in a data-dependent acquisition mode. The orbitrap was set to analyse the survey scans at 60,000 resolution (at *m/z* 400) in the mass range *m/z* 300 to 2000, and the top twenty multiply charged ions in each duty cycle were selected for MS/MS in the LTQ linear ion trap. Charge state filtering, where unassigned precursor ions were not selected for fragmentation, and dynamic exclusion (repeat count, 1; repeat duration, 30 s; exclusion list size, 500) were used. The fragmentation conditions in the LTQ were as follows: normalised collision energy, 40%; activation Q, 0.25; activation time, 10 ms; and minimum ion selection intensity, 500 counts.

The raw data files (ProteomeXchange, https://massive.ucsd.edu, MSV000090853) were processed and quantified using Proteome Discoverer software v1.4 and searched against the UniProt databases, as well as the reverse decoy database (the same database but with all the protein sequences reversed) by using the SEQUEST algorithm. Peptide precursor mass tolerance was set at 10 ppm, and MS/MS tolerance was set at 0.8 Da. Search criteria included the carbamidomethylation of cysteine (+57.0214 Da) as a fixed modification and the oxidation of methionine (+15.9949 Da) as a variable modification. Searches were performed with full tryptic digestion, and a maximum of 1 missed cleavage was allowed. Each match of a spectrum to a peptide was given a score based on how closely the spectrum matched the predicted given peptide sequences. Therefore, any matches to the decoy database were expected to have low scores. The match between the spectrum and the highest-scoring peptide was defined as a peptide-spectrum match (PSM). The PSMs were statistically validated to avoid false positives by using the false discovery rate (FDR)-controlling procedure. In this procedure, scores with an FDR at which there was a > 5% chance that a peptide matched the reversed decoy database were excluded from the final dataset. Peptides with scores between 1% < FDR < 5% were defined as medium confidence peptides, while peptides with scores at an FDR < 1% were defined as high confidence peptides (Tables S1–S3).

### 2.4. Bioinformatic Analyses

The mass spectrometry datasets of the three pollen coat proteomes were tested for significant overrepresentation of gene ontology (GO) terms based on their annotations in the PANTHER (Protein ANalysis THrough Evolutionary Relationships) classification system (Available online: https://www.pantherdb.org, version 13.1, accessed on 19 July 2019). Overrepresentation tests were performed based on the default reference list of *Arabidopsis thaliana* and the complete datasets of GO terms describing biological processes, molecular functions, and cellular components. Overrepresentation tests of *Arabidopsis lyrata* and *Brassica oleracea* were based on the GO annotation of putative orthologues/best hit (based on EnsemblPlants, available online: http://plants.ensembl.org, accessed on 17 June 2018) in the *A. thaliana* genome. Fisher’s exact test with FDR correction was performed. The expression level of pollen coat CRP-encoding genes in different *A. thaliana* tissues were extracted from the TraVa RNA-seq database (Available online: http://travadb.org/, accessed on 17 August 2022) [40,41]. The targets of the analyses were selected based on the classifications in InterPro (ver. 90.0) and previous studies [9,42,43,44,45]. For the detection of codon sites under positive selection, putative orthologous genes were selected from ten species in Brassicaceae: *Arabidopsis thaliana*, *Arabidopsis halleri*, *Arabidopsis lyrata*, *Boechera stricta*, *Brassica oleracea*, *Brassica rapa*, *Brassica napus*, *Capsella grandiflora*, *Capsella rubella*, and *Eutrema salsugineum*. The site-specific selection analysis was processed using the codeml tool in the PAML package (v4.9) [46]. Two pairs of models were used to test whether a proportion of the codon sites was under positive selection: the M1 (nearly neutral) and M2 (positive selection); the M7 (beta); and M8 (beta & ω). The model comparisons were performed using the likelihood ratio test (LRT). The LTR statistic followed the Chi-square distribution. The number of additional parameters in the more complex model determined the degree of freedom (*df*). A twice the log-likelihood (2ΔIn*L*) difference between the two models was compared to the critical value, and the *p*-value was calculated based on the *df* and 2ΔIn*L*.

## 3. Results

### 3.1. Isolation and Proteomic Profiling of the Pollen Coat Proteins from Arabidopsis thaliana, Arabidopsis lyrata, and Brassica oleracea

**Figure 1:**
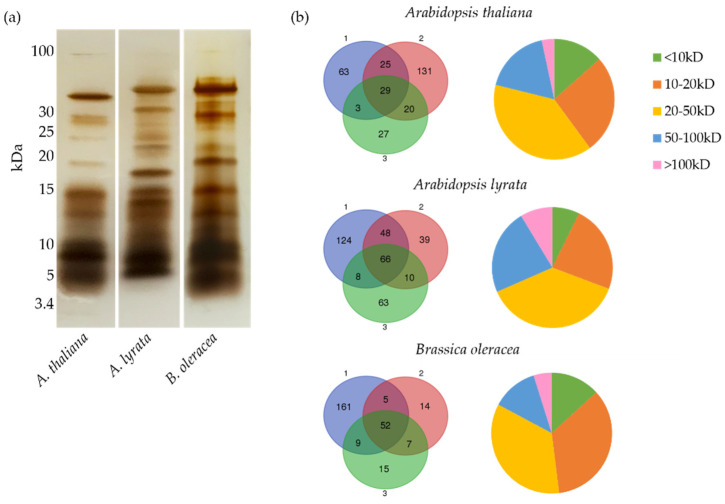
Characterisation of pollen coat protein profiles of *Arabidopsis thaliana*, *Arabidopsis lyrata*, and *Brassica oleracea*. (**a**) Verification of pollen coat protein extracts from *A. thaliana*, *A. lyrata*, and *B. oleracea*. A total of 5% of the total extraction from each preparation was subjected to separation by sodium dodecyl sulphate-polyacrylamide gel electrophoresis (SDS-PAGE), followed by silver staining. (**b**) Overlap of proteins identified in the three replicates (**left panel**) and distribution of protein sizes in each pollen coat gained from three merged proteomic datasets for each species (**right panel**).

Pollen coat extracts were prepared from *Arabidopsis thaliana*, *Arabidopsis lyrata*, and *Brassica oleracea*, and sodium dodecyl sulphate-polyacrylamide gel electrophoresis (SDS-PAGE) analysis confirmed the successful isolation of pollen coat proteins, as well as revealed similar patterns of protein distribution and relative abundance for all three species (Figure 1a). Most conspicuously, all three species displayed abundant protein bands in the 5–20 kDa range (Figure 1a). Despite the overall similarities in the protein profiles obtained, it is noteworthy that the consistency of pollen coat extracts varied between the species following evaporation of the extraction solvent (cyclohexane) on glass slides. For *A. lyrata* and *B. oleracea*, the pollen coat residue was noted to have a honey-like appearance and consistency. In contrast, the pollen coat residue from *A. thaliana* formed a yellow oily crust.

To gain further insights into the protein components of the pollen coat, protein samples extracted from the three species were analysed by LC-MS/MS. Three independent replicates of pollen coat protein extract for each species were analysed to ensure good coverage and a more accurate picture of the pollen coat proteomes. Merged datasets among replicates revealed 298, 358, and 263 unambiguous protein identifications from the pollen coats of *A. thaliana*, *A. lyrata*, and *B. oleracea*, respectively (Tables S1–S6). The relative abundance (estimated based on the percentage of peptide area in each dataset) of proteins identified from the *A. thaliana* pollen coat indicated that the proteins that were shared across more than one replicate were often relatively abundant in the pollen coat (Figure S1). Thus, the lack of substantial overlap among replicates (Figure 1b) was likely due to the naturally low abundance of pollen coat proteins, rather than a result of false identification, which indicated the importance of having sample replicates for the proteomic analyses of scarce materials. The overall analysis of the pollen coat proteomes obtained from the three species revealed profiles with a size range of 5–254 kDa containing a large proportion of small proteins (Tables S4–S6). For *A. thaliana*, *A. lyrata*, and *B. oleracea*, 42%, 31% and 48% of proteins, respectively, had a molecular weight below 20 kDa, (Figure 1b). The overall similarities in the scale of proteins identified and their relative proportions in terms of sizes (Figure 1b) across the three proteomes suggested that the bulk of pollen coat proteins from each species were captured in this study.

### 3.2. Pollen Coat Proteins Are Enriched in Functions Related to Signalling, the Cell Wall, and Lipid Metabolism

**Figure 2:**
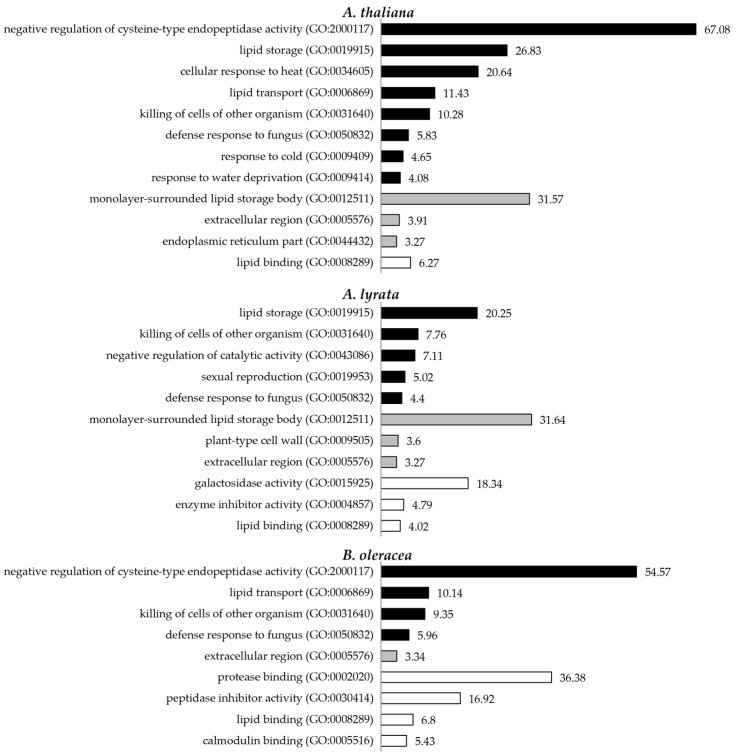
Gene ontology (GO) enrichment analyses of the pollen coat proteomes of *A. thaliana*, *A. lyrata*, and *B. oleracea*. Numbers beside the bars demonstrate the fold enrichment of GO terms over the expected values. Only the categories with fold enrichment >3 and a *p*-value (determined by Fisher’s exact test) <0.05 are shown. Categories with fold enrichment <3 are shown in Figures S2–S4. Black bars indicate biological processes, grey bars indicate cellular components, and white bars indicate molecular functions.

To obtain insights into the functional properties of the pollen coat proteomes, we performed gene ontology (GO) enrichment analyses using 227 annotated proteins from the *A. thaliana* datasets, 326 putative orthologues in *A. thaliana* of the *A. lyrata* datasets, and 183 putative orthologues in *A. thaliana* of the *B. oleracea* datasets. The analyses of pollen coat proteomes obtained for the three species revealed a similar enrichment of GO terms (Figure 2 and Figures S2–S4). For the GO enrichment in the biological process of pollen coat proteomes in *A. thaliana* and *B. oleracea*, the GO term ‘negative regulation of cysteine-type endopeptidase activity’ showed the most significant enrichment (Figure 2, Figure S2 and Figure S4). This resulted from the detection of three cysteine proteinase inhibitors (CYS1, 2 and 6) from the *A. thaliana* pollen coat and three homologues of CYS1, 2, and 4 from the *B. oleracea* pollen coat (Tables S7 and S9). ‘Lipid transport’ was the second most enriched biological process term for *A. thaliana* and *B. oleracea* pollen coat proteomes, while ‘lipid storage’ was the most enriched term for the *A. lyrata* pollen coat proteome. GO terms associated with ‘killing of cells from other organisms’ and ‘defence response to fungus’ were enriched in the pollen coat proteomes of all three species analysed. For the cellular components, the GO terms ‘extracellular region’ or ‘apoplast’ were enriched in all three of the pollen coat proteomes, which corresponded to the nature of the pollen coat localisation of the detected proteins (Figure 2, Tables S7–S9). Some of the proteins identified by the analysis were classed as cytosolic components, which suggested that many tapetum cell-derived proteins were retained in the mature pollen coat following the dissolution of the cell layer during pollen development. The significant enrichment of ‘monolayer-surrounded lipid storage body’ resulted from the detection of multiple oleosin/glycine-rich proteins (GRPs) in the pollen coat of *A. thaliana* and *A. lyrata* (Figure 2, Tables S7 and S8). In terms of molecular function, ‘lipid binding’ was significantly enriched in the pollen coat proteomes of all three species analysed (Figure 2, Tables S7–S9). Overall, overrepresented categories for the three pollen coat proteomes largely overlapped, including GO terms related to lipid metabolism, response to biotic and abiotic stress, and extracellular/apoplastic localisation (Figures S2–S4, Tables S8 and S9).

To achieve further insight into our pollen coat protein profiles, the identified proteins were categorised based on their descriptions associated with biological functions. Protein hits from *A. lyrata* and *B. oleracea* with a description of ‘uncharacterised’, according to the UniProt databases, were categorised based on the description of their best BLAST hits (BLASTP) in *A. thaliana*. Members of protein families associated with the biological functions ‘signal transduction’, ‘lipid metabolism’, ‘cell wall-related’, ‘response to stress’, ‘redox,’ and ‘proteolysis’ were identified in the pollen coats of all three species analysed (Table 1 and Table S10). Proteins associated with ‘signal transduction’, ‘lipid metabolism,’ and ‘cell wall-related’ were the most abundant in number, which covered 63%, 12%, and 8% of proteins classified into the six categories, respectively, which corresponded to the results of GO enrichment analyses. Proteins associated with ‘response to stress’, ‘redox,’ and ‘proteolysis’ covered 7%, 5%, and 5% of the categorised proteins, respectively (Table 1).

In all three Brassicaceae species analysed, we detected proteins known to be important for the biophysical properties of the pollen surface and substantially expanded the members of the protein groups [20,32]. Oleosin-like proteins / glycine-rich proteins (T-oleosins/GRPs) and esterase/lipases (EXLs) were previously identified pollen coat components that are considered crucial for maintaining the conduit for water through the pollen coat during pollen hydration [20,21]. This analysis identified 24 T-oleosin GRPs and 12 EXLs that further confirmed their importance for the initiation of pollination by contributing to the biophysiological nature of the pollen coat. Some cell-wall degrading enzymes, such as xylanases and β-glucanases, have been previously found to be pollen coat components in grasses [30,47,48]. Enzymes that hydrolyse or modify cell wall components, such as pectinase, galactosidase, glucosidase, and xyloglucan endotransglucosylase, were also found to be abundant in the pollen coat proteomes (Table 1 and Table S10). These proteins may represent remnants of enzymes involved in the degradation of the tapetum cell wall during pollen development, but could also specifically function in facilitating penetration of the sigmatic cell wall by the pollen tube at the pollen–stigma interface [49]. In particular, the detection of various pectin-related enzymes, such as pectinases, pectin esterases, pectin lyases, and pectin methylesterase inhibitors (PMEIs), in the pollen coat suggests the involvement of the pollen coat in facilitating stigmatic cell wall loosening and promoting pollen tube extension during the first stage of germination following compatible pollination.

In addition to CRPs (described below), we identified signalling proteins that were previously not known to be associated with the pollen coat. Self-incompatibility protein homologues (SPHs) belong to a large family of proteins related to the *Papaver rhoeas* stigma *S*-determinant (PrsS), which is the female determinant of the *Papaver* SI system [50,51]. A total of 17 SPHs were detected in our pollen coat proteomes (Table 1 and Table S10). Although SPHs have similar features to CRPs, they generally have fewer cysteine residues, are larger, and, thus, are treated as a separate category [52]. Since the discovery of PrsS in *Papaver*, more than 2000 SPHs and SPH domain-containing proteins have been found in more than 70 species, which mostly include plants but also include several fungi and metazoans [52]. More than 100 SPHs have been identified from *A. thaliana*, a self-compatible species, mainly in floral tissues, with several in developing leaves [53,54]. This suggests that SPHs are likely to be involved in multiple signalling pathways.

Our pollen coat proteomic analyses also discovered proteins containing carbohydrate-binding domains. Cysteine-rich repeat secretory proteins (CRRSPs) belong to a superfamily of proteins containing domain(s) with a conserved cysteine-rich repeat (CRR) motif CX(8)CXXC, with a Domain of Unknown Function 26 (DUF26; Gnk2 or Stress-antifungal) [55,56]. CRRSPs possess an N-terminal signal peptide and two copies of DUF26. Two CRRSPs, CRRSP18 and CRRSP41, were previously detected in the *A. thaliana* pollen coat [32]. In our study, 28 CRRSPs were detected in the pollen coat across the three species analysed (Table 1 and Table S10). The extracellular double-DUF26 domain has been revealed to be responsible for signal perception and potential redox sensing [57,58,59]. Structural analyses revealed the DUF26 to be related to fungal lectin, with some members possessing carbohydrate binding activities and providing resistance against fungal pathogens, which suggest a common evolutionary origin between plant DUF26 and eukaryotic lectins as carbohydrate recognition modules [56,60]. Another intriguing discovery was that D-galactoside/L-rhamnose binding SUEL (sea urchin egg lectin) domain-containing proteins were detected in the pollen coat. SUEL-related lectins belong to a widely distributed superfamily of proteins containing a carbohydrate-recognition domain (CRD) that is structurally similar to SUEL (reviewed in [61]). Some plant β-galactosidases (BGAL) have also been found to contain this domain at the C-terminus [62]. Our proteomes detected 10 D-galactoside/L-rhamnose-binding SUEL lectin proteins with a signal peptide across three species and one SUEL-type lectin domain containing β-galactosidase in *A. lyrata* (BGAL8) (Table 1 and Table S10). SUELs have been found to be specifically localised to the peripheral layer of embryonic cells in marine animals, and some specifically bind to D-galactoside or L-rhamnose components of the bacterial cell wall, which suggests their potential roles in mediating intercellular interactions or innate immunity (reviewed in [61]). Pollen–pistil interactions are often regulated by protein–protein interactions, many of which have CRPs as ligands (reviewed in [12,28]). Considering the importance of sugar molecules in cell–cell communication, our discovery of pollen coat proteins possessing carbohydrate-binding domains suggests an alternative model of pollen–stigma communication via protein–carbohydrate interaction. In summary, the enrichment in functions related to signal transduction, lipid metabolism, and cell wall modification within the proteomes is entirely consistent with the lipidic nature of the pollen coat, which functions in terms of providing protection of the male gametophyte and as an essential mediator of the earliest stages of sexual reproduction.

### 3.3. Small CRPs Are Highly Represented in the Pollen Coat of Members of the Brassicaceae

Small CRPs are an important and diverse group of proteins in plants that typically possess an N-terminal signal peptide that targets them for secretion. Many CRPs have been identified as signalling and antimicrobial proteins; however, most have not been functionally characterised. The classification of CRPs is determined by their conserved cysteine residues that typically form disulphide bridges, which stabilise the overall structural fold of the protein. Our proteomic analyses of the pollen coat revealed a total of 162 small CRPs across the three analysed species (Table 1 and Tables S11–S13). A total of 157 of these CRPs fell into five classes: the pollen coat protein A class (PCP-A), which includes defensin-like proteins (DEFLs) and low-molecular-weight cysteine-rich proteins (LCRs); the pollen coat protein B class (PCP-B); *S*-locus cysteine-rich-like proteins (SCRLs); non-specific lipid transfer proteins (nsLTPs); and gibberellic acid-stimulated Arabidopsis (GASAs). Five identified small CRPs were not categorised into any of these classes based on their cysteine patterns, including an early culture abundant 1 (ECA1)-like gametogenesis-related family protein in *A. thaliana* and *B. oleracea*, respectively, a rapid alkalization factor (RALF)-like protein in *B. oleracea*, a prolamin-like domain-containing protein in *B. oleracea*, and an uncharacterised protein (Table 2, Table S11 and Table S13).

CRPs have been previously identified as important players in reproductive signalling. Several PCP-As have been implicated as important signalling regulators of pollination in *Brassica* [7,24,36,38,63,64]. CRPs with the same pattern of cysteine residues in *A. thaliana* were previously categorised as DEFLs [65] or LCRs [42]. In our proteomic analyses, 72 proteins with the same cysteine pattern as PCP-A/ DEFL/ LCR were detected across the three species. SCRLs possess the same pattern of cysteine residues as SCR, which is the male determinant of sporophytic self-incompatibility (SSI) in *Brassica* [8,22,66]. We identified 34 SCRLs in the pollen coats across the three species and captured 10 of the 28 known SCRLs in *A. thaliana* [42]. PCP-Bs represent the first pollen coat proteins discovered as key regulators of basal pollen–stigma compatibility in *A. thaliana*, with *PCP-B* mutant pollen having impaired hydration on compatible stigmas. PCP-Bs act as negative regulators of stigmatic ROS via interaction with stigmatic transmembrane receptors, which reduce ROS thus facilitating pollen hydration [9,25]. Our proteomic analyses detected PCP-Bα, PCP-Bγ, and PCP-Bδ, which are three of the four previously identified PCP-Bs in *A. thaliana*, as well as a putative orthologue of PCP-Bγ in *A. lyrata* and putative orthologues of PCP-Bγ and PCP-Bδ in *B. oleracea*. Additionally, we also identified a previously uncharacterised PCP-B, PCP-Bε (Q1G3R6, At2g41415), in *A. thaliana* and its putative orthologue (D7LH69) in *A. lyrata* (Tables S11 and S12). The non-specific lipid transfer proteins (nsLTPs) belong to a large protein family involved in diverse aspects of plant development and reproduction (reviewed in [67]). A member of the nsLTP group, stigma/style cysteine-rich adhesin (SCA), is specifically expressed in the stigma and style of *Lilium longiflorum* [68]. A functional study of AtLTP5, an SCA-like protein in *A. thaliana*, suggests its involvement in pollen tube growth guidance in the pistil transmitting tract [69]. We identified 37 nsLTPs across the three species analysed. A total of 10 of the 79 Arabidopsis nsLTPs reported by Edstam et al. [45] were detected in *A. thaliana*. A9, a tapetum-specific LTP involved in pollen wall development [70,71], was also detected. GASAs belong to a class of CRPs that have been found to be involved in various processes relating to plant development, including plant growth, seed development, flowering, and responses to abiotic and biotic stresses [43,44,72,73,74,75]. A total of 14 GASA-encoding genes have been identified in *A. thaliana*, with GASA4 having been shown to be involved in gibberellic acid responses related to flowering and seed germination [43,72]. Two *A. thaliana* GASAs (GASA 10 and 11) and their orthologues were detected in our pollen coat proteomes across the three species. Although the functions of the majority of the CRPs detected in our proteomes are unknown, the involvement of members of these CRP families in reproductive and developmental signalling suggests that many of these proteins may be playing important roles in early pollen–stigma signalling.

**Figure 3:**
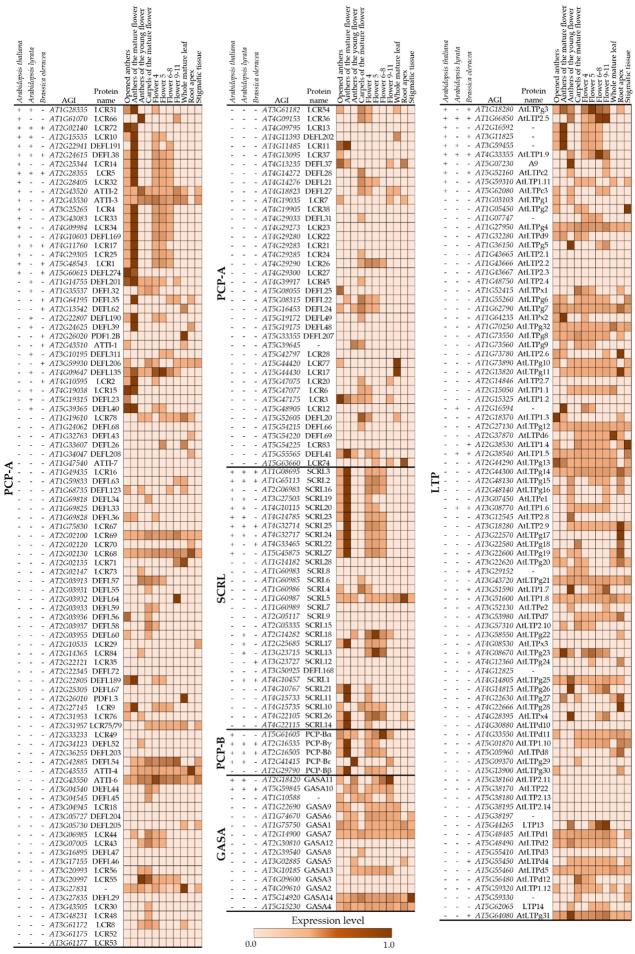
RNA-seq analyses from *A. thaliana* of the genes encoding five cysteine-rich protein (CRP) families identified from pollen coat proteomes. The ‘+’ and ‘-’ beside the Arabidopsis gene initiative (AGI) locus code indicate whether the protein was identified in the pollen coat proteomes in this study. Coloured boxes indicate relative gene transcript levels that were processed as normalised read counts by the median-of-ratio (Med) method [77] in opened anthers, anthers of the mature flower (before opening), anthers of the young flower, carpels of the mature flower, young flowers (Flower 4–11), whole mature leaf, roots apex, and stigmatic tissue (Available online: http://travadb.org, accessed on 17 August 2022).

To gain further insights into the developmental origins of pollen coat CRPs and how they relate to their wider gene families, we analysed the expression patterns of all known *A. thaliana* genes belonging to the five pollen coat CRP families by mining the TraVa RNA-seq database (Available online: http://travadb.org/, accessed on 17 August 2022) [40,41]. The expression profiles showed genes encoding one PCP-A/DEFL/LCR, one PCP-B, and six LTPs that have high expression levels in anthers from young flowers (stage 9, [76]). A further three PCP-As, one PCP-B, two SCRLs, five LTPs and one GASA were highly expressed in young flower buds (flower stage 4–11, http://travadb.org/),(accessed on 17 August 2022) which together suggest their possible origin from tapetum cells and potential roles in developmental signalling during early anther/pollen development, or their roles as regulators of the pollen–stigma interaction (Figure 3). Genes encoding 31 PCP-A/DEFL/LCRs, 3 PCP-Bs, 15 SCRLs, and 10 LTPs were specifically expressed in mature anthers (opened anthers or anthers before dehiscence at stage 13, [76]), with very little to no expression in leaves, root tips, or stigmatic tissue. This strongly suggests that these CRPs are expressed in pollen grains and secreted to the pollen surface, as has previously been reported for a number of members of the PCP-A, PCP-B, and SCRL protein families [7,8,9]. No GASA-encoding genes were found to be specifically expressed in mature anthers. Among the mature anther-specific CRPs, 24 PCP-A/DEFL/LCRs, 2 PCP-Bs, 11 SCRLs, and 6 LTPs or their putative orthologs were detected from at least one species in our pollen coat proteomes (Figure 3), which demonstrated that our datasets aligned well with published expression profiles. Though the functions of most of these small peptides are not clear, the detection of abundant numbers of CRPs from the pollen coats and their mature anther-specific expression patterns suggest their potential roles in pollen–stigma signalling.

### 3.4. Evidence of Positive Selection in Regions of CRPs Identified in the Pollen Coat Proteomes

**Figure 4:**
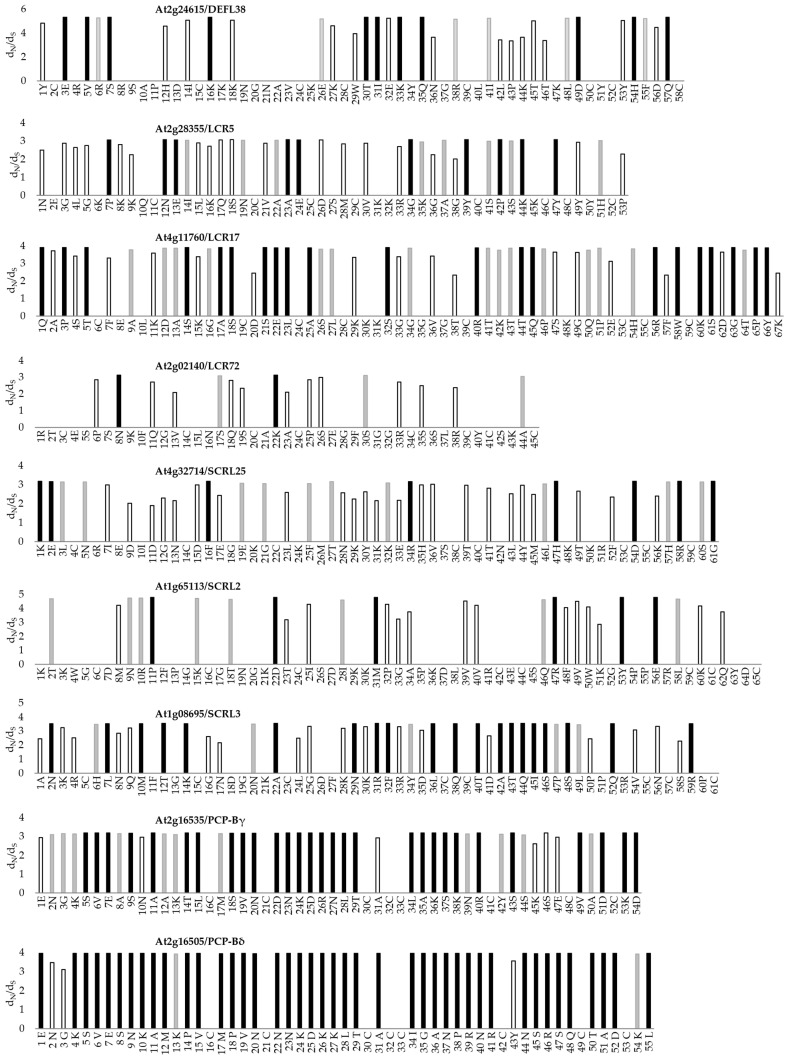
Positive selection and ω (d_N_/d_S_) of codon sites with a posterior mean ω > 1 of nine pollen coat cysteine-rich proteins (CRPs). Site-specific selection analysis was based on model M8 in the codeml programme of the PAML package [46]. The amino acid residues are shown below the *x*-axis. The ω values with a Bayesian posterior probability greater than 99% are represented by black bars, ω values with a Bayesian posterior probability greater than 95% are represented by grey bars, and ω values with a Bayesian posterior probability less than 95% are represented by empty bars.

Adaptive evolution is considered to be the common feature of genes that mediate sexual reproduction [78]. Many of these genes have been identified as ‘speciation genes’, which contribute to the formation of reproductive barriers by reducing the amount of gene flow between populations (reviewed in [79]). We considered that some of the CRPs identified in the pollen coat proteomes could potentially be encoded by ‘speciation genes’ that undergo rapid diversification and evolve under positive selection to adapt to their interacting receptors. We performed evolutionary analyses to examine if the CRP-encoding genes showed evidence of positive selection (Figure 4), and we focused on the 12 pollen coat CRPs with orthologues that were shared amongst all the three species analysed (Figure 3). To estimate the selection pressure on the sites of each CRP-encoding gene, the ratio of the number of nonsynonymous substitutions / synonymous substitutions per possible non-synonymous codon site ω (d_N_/d_S_) was calculated using multiple methods in the codeml programme in PAML [46]. The phylogeny and codon alignment of putative orthologous genes were analysed using three models (M0, M7 and M8) within the codeml programme [46]. Model M0 performed the same ω (d_N_/d_S_) ratio, and assumed that all the sites were under the same selection pressure. By using this method, only two CRPs (PCP-Bγ and PCP-Bδ) showed an average ω (d_N_/d_S_) >1 (Table 3). To demonstrate the variation of selection force among sites and to detect sites with ω > 1, we compared model M7 (beta distribution, ten rates confine ω to the interval of 0 to 1) and model M8 (beta distribution also allows ω = 1) using a likelihood ratio test (LRT) for each gene [80,81]. For the genes with a result where model M7 was rejected in favour of model M8, M8 was carried out to identify sites under positive selection using the Bayes empirical Bayes (BEB) method [82] (Table 3). The analyses assumed that the sites with ω > 1 and a Bayesian posterior probability ≥99% were likely to be under positive selection (Table 3, Figure 4). For 9 out of 12 analysed CRPs, the positive selection model M8 appeared to fit the data significantly better than the null model M7 (Table 3). Figure 4 demonstrates the ω (d_N_/d_S_) ratio for each site calculated under M8 that was greater than 1, which illustrates the CRP-encoding amino acid sites that had approximate means of posterior distribution ω > 1. The conserved cysteine residues showed no evidence of positive selection, which corresponded to their function of maintaining the secondary structures of the molecules by forming disulphide bonds. These results provide evidence of different selection pressures on amino acid sites along the CRP sequences and positive selection on the sites between cysteines.

## 4. Discussion

### 4.1. Significant Expansion of the Known Pollen Coat Proteome in the Brassicaceae

**Figure 5:**
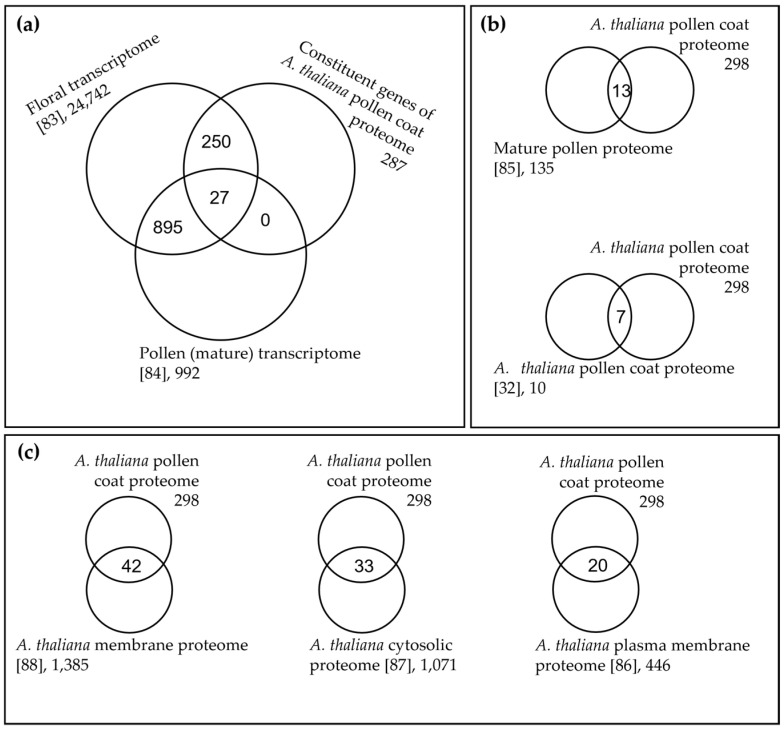
Overlaps of *Arabidopsis thaliana* pollen coat proteome or constituent genes with previously reported proteomes and transcriptomes. (**a**), Overlaps among constituent genes of *A. thaliana* pollen coat proteomes from this study, the floral transcriptome [83], and the pollen transcriptome [84]. (**b**), Overlaps of *A. thaliana* pollen coat proteomes from this study with a mature pollen proteome [85] and previously reported *A. thaliana* pollen coat proteomes [32], respectively. (**c**), Overlaps of *A. thaliana* pollen coat proteomes from this study with previously reported *A. thaliana* membranes and cytosolic proteomes, respectively [86,87,88].

When compared with previous studies, our proteomic analyses achieved a significant improvement in the number of proteins identified in the pollen coat amongst three members of the Brassicaceae [31,32]. With respect to our *A. thaliana* proteome, 277 out of the 287 constituent genes of the pollen coat proteins identified were also included in the published floral transcriptome [83]. Furthermore, only 27 of these are included in the mature pollen transcriptome (Figure 5a) [84], which suggests that the majority of proteins present in the pollen coat are expressed just before anther dehiscence or derived from the anther tapetum during pollen development. Only a very small proportion (4%) of proteins identified in our *A. thaliana* pollen coat proteome overlapped with the previously published whole mature pollen proteome, which was likely predominantly composed of intracellular proteins [85] (Figure 5b). Taken together, these data validate the precision of the techniques used in this study to only isolate proteins from the pollen coat domain. Sixty-six proteins from our *A. thaliana* pollen coat proteome were previously identified from at least one of the membrane or cytosolic proteomes for *A. thaliana* (Figure 5c, Table S14) [86,87,88], which may be the result of the degeneration of tapetal cells or potentially minor contamination from broken cells during pollen coat isolation. Our proteomic analyses of the pollen coat from three intensively studied species in the Brassicaceae provided a dramatically improved coverage of the pollen coat protein constituents, especially ones that have very low molecular weights (<10 kDa)—these have never been detected from any other published pollen coat protein profiles to date.

### 4.2. Enrichment of the Proteins Associated with Signal Transduction, Cell Wall, and Lipid Metabolism in Pollen Coat Proteomes

The observation that large numbers of the pollen coat proteins identified in this study were categorised into the GO functional groups ‘signal transduction’, ‘lipid metabolism,’ and ‘cell wall-related’ reflects the biosynthetic processes underlying pollen wall and pollen coat development, as well as the function of the pollen coat in pollination. Amongst members of the Brassicaceae, the lipidic pollen coat is largely derived from the tapetum, which is a specialised, highly secretory cell layer lining the anther locule that undergoes programmed cell death (PCD) late in pollen development. Following tapetal dissolution, released cellular contents that are rich in lipids and proteins are deposited on the outer exine layer of the maturing pollen grains [3]. The protein profiles derived from this study are consistent with the known cellular and molecular events that occur during other developments, including the formation of the pollen wall, the pollen coat, and the degradation of the tapetum (reviewed in [89,90]). Not only are lipids crucial precursors for the biosynthesis of the sporopollenin exine layer of pollen, but they are also essential for the biophysical properties of the pollen coat that are central to successful pollination [16,17,18]. In addition to the previously studied pollen coat factors known to impact pollination, such as oleosins /glycine-rich proteins (GRPs) and GDSL esterase/lipases [20,21], the identification of additional proteins related to lipid metabolism provides new candidates for further investigations into the molecular mediators of pollen coat formation and function.

Pollen germination and tube growth through the stigmatic tissue requires the pollen tube to breach several layers of the pollen wall, and penetrate the stigmatic papilla cuticle and its outer cell wall [91,92]. As the primary interaction domain, the pollen coat may thus carry and deliver enzymes that promote changes in pollen or papilla cell wall chemistry to facilitate the penetration of the pollen tube [93]. Our identification of numerous cell wall modifying enzymes, including pectinase, glucosidase, galactosidase, and xyloglucan endotransglucosylase, strongly suggests that the pollen coat does contribute to this process, though it is likely that proteins secreted by the growing pollen tube and the papilla cell itself are also involved.

The recognition and acceptance of compatible pollen, or rejection of incompatible pollen, by the highly discriminative dry stigma requires strict intercellular signalling processes. Interestingly, all the pollen coat proteins identified to date that are known or strongly suspected to mediate this process are small cysteine-rich proteins (CRPs). For example, the pollen coat CRP *S*-locus cysteine-rich protein (SCR) acts as the male determinant of SSI in *Brassica*, which, through interacting with the stigmatic female determinant *S*-receptor kinase (SRK), brings about self-pollen rejection [8,22,66]. Other pollen coat proteins in *Brassica* belonging to the PCP-A class, including *S*-locus related-binding protein (SLR-BP1) and PCP-A1, interact with the stigma-specific cell wall proteins SLR1 and *S*-locus glycoprotein (SLG), respectively, though their exact function remains unclear [7,24]. Furthermore, cysteine-rich PCP-B proteins are known to play a role in the regulation of pollen hydration in *A. thaliana* [9,23,25]. Our proteomic profiling of the pollen coat revealed strikingly large numbers of small CRPs of unknown function that fell into five main protein families (PCP-A/DEFLs/LCRs, PCP-Bs, SCRLs, nsLTPs, and GASAs). Across these families, 157 CRPs were identified in the three species studied, with 45 being found in the *A. thaliana* pollen coat. Within these families, protein polymorphism is generally high, which suggests sequence diversification, followed by the maintenance of protein variants. It is tempting to speculate that such diversity reflects the continual evolution of factors that regulate compatibility between mating partners, as has been reported for vertebrate reproductive proteins [94]. Taken together, the broad analysis of the data from all three proteomic profiles reinforces the importance of the pollen coat as a domain rich in factors that are likely to play central roles in early pollination events.

### 4.3. Enrichment of CRPs in the Pollen Coat Provides Insights into the Evolutionary Link between CRP Signalling during Reproduction and Defence

Sexual plant reproduction and plant defence response signalling systems share a common principle: to distinguish ‘self’ from ‘non-self’. Accumulating evidence suggests that CRP signalling involved in plant reproduction probably evolved from an ancient plant defence system. Several small CRPs derived from plant pathogens have recently been found to act as pathogen effectors that manipulate plant immunity [95,96,97,98,99,100]. Comparisons of the morphology and molecular mechanisms of plant defence responses to plant reproductive signalling demonstrate intriguing similarities [101,102,103,104]. Both systems utilise ligand receptor interactions to establish the recognition module, while downstream signalling in cells triggered by the ligand, often by small CRPs, share conserved mechanisms, such as ion influx, ROS production, and PCD (reviewed in [104]). Among the 825 members of CRPs in *A. thaliana* [44], over 300 were originally annotated and defined as defensin-like proteins (DEFLs) based on their N-terminal signal peptide, γ-core, and a cysteine-stabilised αβ (CSαβ) motif that forms a thermostable pseudo-cyclic structure [65,105]. Functional studies on members of the DEFL family initially reported their pivotal role in the innate immune system of plants by acting in defence against pathogens (reviewed in [106]). Most CRPs involved in signalling during plant reproduction belong to the DEFL family [26,27,28]. Some of them have been found to have dual functions and are involved in both reproductive signalling and defence response in plants [107,108,109]. Remarkably, a receptor-like kinase, FERONIA (FER), functions through interacting with CRPs in both plant reproductive and plant defence signalling [25,110,111,112]. A systematic transcriptomic study on the reproductive and immune responses in the pistil of *Arabidopsis* species revealed that the genes encoding CRP subgroups CRP0570 (PCPAL/DEFL/LCR) and CRP0830 (SCRL) were down-regulated during both pollination and fungal infection in *A. thaliana* and *A. halleri*, which suggests that they might also function to protect reproductive tissues from pathogen attacks [113]. The five main CRP classes identified in our *A. thaliana*, *A. lyrata*, and *B. oleracea* pollen coat proteomes accounted for 15%, 13%, and 25% of identified proteins, respectively. These proportions are dramatically larger than the estimation that genes encoding CRPs account for 2–3% of all genes in *A. thaliana* and *Oryza sativa* [44] and corresponds to the previous observation that CRPs are overrepresented in reproductive structures [44,104,114]. Taken together, our detection of numerous CRPs in the pollen coat, which are frequently exclusively expressed in mature anthers, adds further weight to the hypothesis that reproductive CRPs have evolved from antimicrobial proteins, probably via sub- or neo-functionalization following gene duplication events.

### 4.4. Positive Selection on CRP-Encoding Genes Suggests Roles in Contributing to Species Barrier Formation

Genome-wide comparative sequencing studies in multiple species have shown that genes mediating processes in sexual reproduction evolve more rapidly than other genes, which contributes to reproductive isolation (reviewed in [115]). The rapid divergence of reproductive proteins is likely to be promoted by adaptive evolution [78]. Many previously published reports that have investigated the modes of selection for gene families, including those encoding members of protein families identified from this study, were based on pairwise comparison methodologies, which assumed that the selective pressure on each codon site was the same. Pairwise evolutionary analyses of more than 300 mature DEFLs in *A. thaliana* demonstrated 18 pairwise comparisons with evidence of divergent selection (mean nonsynonymous/synonymous rate (*Ka/Ks)* values > 1) [65]. A relatively rapid evolutionary process (mean *Ka/Ks* values > 1) was also detected in LTPs in rice and wheat by pairwise comparisons within and between different LTP types [116]. The same approach was taken to study the evolution of pollen-specific T-oleosins/GRPs amongst members of the Brassicaceae, which also provided some evidence of rapid evolution [117,118]. Analyses of the evolution of reproductive genes in *Solanum* revealed elevated evolutionary rates in reproductive proteins encoded by female-specific loci [119]. However, the pairwise comparison method does not reflect the variation in selection force within a sequence, which may then result in signals of adaptive divergence among domains or sites being missed. The analyses of several *CRP* gene families in *Pyrus bretschneideri* using codeml in PAML revealed variation in positive selection amongst amino acid sites [120]. Analyses of the evolution of the *S*-locus region in *Arabidopsis* and its relatives have provided evidence of variation in positive selection among sites within *SCR*, *SRK*, and *ARK3* (*A. thaliana receptor kinase 3*) [121]. Our evolutionary study of a subset of CRPs that are shared across the three pollen coat proteomes of this study has revealed a similar story—significant variation in selective pressure was detected among codon sites. It is also noteworthy that all the conserved cysteine residues were under purifying selection (Figure 4). This is not surprising, as these residues are crucial for the maintenance of CRP secondary structures around which variations arising in the primary sequence can lead to the evolution of polymorphisms that could underlie mating partner specificity or functional diversification. Thus, the discovery of positively selected sites in genes encoding the nine pollen coat CRPs in this study demonstrates their potential involvement in processes that contribute to reproductive isolation by acting as compatibility recognition factors at the pollen–stigma interface. Taken together with the examples reported for genes at the *S*-locus, our data also strongly support the hypothesis that the evolution of reproductive proteins is driven by selective pressure to establish reproductive barriers through their interactions with molecular targets [121]. Considering the large numbers and diversity of CRPs identified in this and other studies that have potential roles in plant reproduction and defence, analyses directed at the detection of sites under positive selection may be a fruitful strategy to identify those proteins that play a central role in cell–cell recognition and communication.

## 5. Conclusions

As the outermost layer of the pollen grain, the pollen coat plays key roles in protection of the male gametophyte, pollen–stigma recognition, pollen adhesion, hydration, pollen germination, and stigmatic penetration. Prior to this study, very few pollen coat proteins had been identified directly from the pollen coat, and even fewer had been functionally characterised. The proteomic analyses reported in this study have revealed the *Brassica*/*Arabidopsis* pollen coat to contain a remarkable number and diversity of proteins that reflect the functional importance of this pollen domain in reproduction. In particular, the plethora of CRPs identified was a striking feature across all three species studied. Many of these CRPs are encoded by highly diverse gene families, with some members already known to function as signalling factors in sexual plant reproduction. Thus, it is tempting to speculate that many of the proteins of unknown function identified in this study function in pollen–stigma signalling. Indeed, the evidence of positive selection amongst a number of CRPs uncovered in this study strongly suggests they could be contributing to the establishment of reproductive barriers. The variation in selective force across sites within a sequence also provided tantalising insight into the protein domains that may be particularly important in defining pollen–stigma specificity. The highly duplicated and diversified genes encoding pollen coat CRPs likely provide the evolutionary raw material that enables male gametophyte competition early in pollination at the point of pollen hydration and germination. Thus, factors carried by the pollen coat will contribute to the formation of reproductive barriers that can drive speciation. What is emerging from the few functional studies carried out to date, and the data presented here, is that multiple pollen coat factors simultaneously contribute to the regulation of pollen–stigma compatibility and thus the success, or otherwise, of individual pollen grains. Our proteomic analyses have uncovered a ‘reservoir’ of potential protein regulators in plant reproductive signalling and, thus, provide a solid platform from which to launch future research on pollen coat function and the evolution of breeding systems.

## Figures and Tables

**Table 1:** Categories of protein families identified in the pollen coat from *A. thaliana (A. t)*, *A. lyrata (A. l)*, and *B. oleracea (B. o)*.

Protein Description		*A. t*	*A. l*	*B. o*
**Signal transduction**	14-3-3-like protein	4	2	0
	Cysteine-rich repeat secretory protein (CRRSP)	7	7	14
	F-box protein	2	5	1
	D-galactoside/L-rhamnose binding SUEL protein	3	3	5
	Kinase	6	16	14
	S-protein homolog (SPH)	6	8	3
	Small cysteine-rich protein (CRP)	47	45	70
**Lipid metabolism**	Acyl-CoA-binding protein	1	1	2
	Acyltransferase	2	1	0
	esterase/lipase (EXL)	5	4	3
	Oleosin/Glycine-rich protein (GRP)	8	6	10
	Glycosyltransferase	1	2	0
	Lipid binding protein	1	1	2
	Sterol carrier protein	1	0	1
**Cell wall-related**	Acetylglucosaminyltransferase	0	1	1
	Galactosidase	1	5	1
	Glucosidase	1	1	0
	Pectin acetylesterase	0	1	1
	Pectin lyase-like protein	0	5	1
	Pectin methylesterase inhibitor (PMEI)	1	6	3
	Pectinase	1	0	1
	Pectinesterase	1	1	0
	Xyloglucan endotransglucosylase	0	1	1
**Response to stress**	Heat shock cognate protein	0	1	2
	Late embryogenesis abundant protein (LEA) family protein	4	5	2
	Protein cold-regulated	2	2	0
	Proteinase inhibitor	4	3	4
**Redox**	FAD-linked oxidase	1	2	0
	Galactose oxidase	0	1	1
	Glutaredoxin	2	2	1
	Peroxidase	1	6	4
	Peroxygenase	1	2	0
**Proteolysis**	Aspartic proteinase	1	1	1
	Carboxypeptidase	2	2	2
	Cysteine proteinase	2	2	0
	Protease	1	2	1
	Proteasome	2	0	1

**Table 2:** Cysteine-rich protein (CRP) classes shared among the pollen coat proteomes of *Arabidopsis thaliana*, *Arabidopsis lyrata*, and *Brassica oleracea*. PCP-A, pollen coat protein A; DEFL, defensin-like protein; LCR, low-molecular-weight cysteine-rich protein; PCP-B(L), pollen coat protein B (-like); SCR(L), *S*-locus cysteine-rich (-like) protein; nsLTP, non-specific lipid transfer protein; GASA, gibberellic acid-stimulated Arabidopsis. The subgroup identifiers are assigned based on Silverstein et al. [44]. The numbers of CRPs belonging to each class were based on merged datasets among three replicates of each pollen coat proteome. Letter ‘C’ represents conserved cysteine residues. Letter ‘X’ represents any amino acid. Numbers in brackets represent the range of residue numbers between cysteines.

CRP Classes (Subgroup Identifiers)	Species	No. of CRPs	Sizes	Cysteine Patterns
PCP-A/DEFL/LCR (CRP0000, 0260, 0520, 0560, 0570, 0580, 0650, 0700, 0710, 0960)	*A. thaliana*	19	76–90	CX(5-12)CX(4-8)CXXXCX(9-16)CX(3-14)CXCX(0-5)C
	*A. lyrata*	21	53–96	CX(7-16)CX(4-8)CXXXCX(9-16)CX(4-13)CXC(1-8)C
	*B. oleracea*	32	52–209	CX(3-21)CX(4-11)CXXXCX(9-15)CX(4-12)CXCX(1-6)C
PCP-B/PCP-BL (CRP5460, 5500, 5515)	*A. thaliana*	4	74–82	CXXXXCX(7-8)CXCCX(6-8)CX(6)CXXXC
	*A. lyrata*	2	74–77	CXXXXCX(7-8)CXCCX(6-8)CX(6)CXXXC
	*B. oleracea*	2	66–134	CX(4-10)CX(8)CXCCX(6-9)CX(6)CXXXC
SCR/SCRL (CRP0830)	*A. thaliana*	10	87–98	CX(9)CX(7)CX(13-19)CXCX(11-15)CXCX(3-7)C
	*A. lyrata*	13	79–105	CX(9-10)CX(7-10)CX(13-19)CXCX(10-16)CXCX(3-7)C
	*B. oleracea*	11	73–108	CX(9-10)CX(7-8)CX(13-23)CXCX(11-27)CXCX(3-7)C
nsLTP (CRP3860, 4000, 4380, 4670, 4820, 4900)	*A. thaliana*	10	91–180	CX(6-9)CX(13-16)CCX(8-19)CXCX(12-25)CX(6-15)C
	*A. lyrata*	7	93–119	CX(6-9)CX(12-16)CCX(8-19)CXCX(17-24)CX(6-15)C
	*B. oleracea*	20	90–265	CX(6-9)CX(13-16)CCX(8-19)CXCX(12-25)CX(6-13)C
GASA (CRP2700)	*A. thaliana*	2	89–94	CXXXCXXXCX(8)CXXXCXXCCXXCX(1-2)CX(11)CXCX(12)C
	*A. lyrata*	2	89–94	CXXXCXXXCX(8)CXXXCXXCCXXCX(1-2)CX(11)CXCX(12)C
	*B. oleracea*	2	89–91	CXXXCXXXCX(8)CXXXCXXCCXXCXXCX(11)CXCX(12)C
Others (CRP1850, 2865, 3600, 3670)	*A. thaliana*	2	104–140	-
	*A. lyrata*	0	-	-
	*B. oleracea*	3	73–165	-

**Table 3:** Evidence of positive selection on the codon sites of genes expressing cysteine-rich proteins (CRPs) shared among three pollen coat proteomes. M0 results showed the average d_N_/d_S_ ratio among all sites and lineages. 2ΔInL (M7 vs. M8) and its *p*-value indicate the results of the likelihood ratio test (LRT). * *p* < 0.05, ** *p* < 0.001, *** *p* < 0.0001. Estimated parameters (M8) show the proportion of amino acid predicted to be under adaptive evolution and corresponding d_N_/d_S_ ratio from model M8. Positive selected sites are amino acids under positive selection with Bayesian posterior *p* > 0.99 based on model M8 using the Bayes empirical Bayes (BEB) method. NA indicates not applicable, because there was no significant difference between the positive selection model and the neutral model. DEFL, (putative) defensin-like protein; LCR, low-molecular-weight cysteine-rich protein; ESFL, embryo surrounding factor 1-like protein; SCR(L), *S*-locus cysteine-rich (-like) protein; PCP-B, pollen coat protein B; nsLTP, non-specific lipid-transfer protein; GASA, gibberellic acid-stimulated Arabidopsis. Ns, number of sequences in the analysis. LA, numbers of codons in the alignment.

Accession (Description)	Gene Locus	Ns/ LA	d_N_/d_S_ (M0)	2ΔInL (M7 vs. M8)	Estimated Parameters (M8)	Positively Selected Sites
**Q2V462** **(** **DEFL38)**	*AT2G24615*	8/87	0.845	39.059 ***	13.4% (ω = 3.217)	23E, 25V, 29S, 36K, 50T, 51I, 53K, 55Q, 70D, 75H, 83Q,
**Q8S8H3 (DEFL149/LCR5)**	*AT2G28355*	9/62	0.885	16.300 **	19.1% (ω = 2.182)	7P, 12N, 13E, 19Q, 20S, 25A, 26E, 28D, 36G, 41Y, 46P, 53K, 55Y
**Q9T0E3 (DEFL151/LCR17)**	*AT4G11760*	9/84	0.825	35.276 ***	21.0% (ω = 2.063)	4Q, 6P, 8T, 21S, 24A, 25S, 34S, 35E, 36L, 38A, 45S, 53R, 57T, 58Q, 73R, 75W, 77K, 78S, 80G, 82P, 83Y
**Q9ZUL8 (DEFL10/LCR72)**	*AT2G02140*	10/56	0.433	7.191 *	9.9% (ω = 1.962)	16N, 30K
**P82644 (DEFL231/SCRL25)**	*AT4G32714*	10/106	0.804	18.579 ***	10.8% (ω = 2.423)	32K, 33E, 47F, 66R, 79H, 86D, 90R, 96G
**P82621 (DEFL226/SCRL2)**	*AT1G65113*	9/65	0.860	29.623 ***	5.9% (ω = 4.293)	11P, 22D, 31M, 47R, 53Y, 56E
**P82622 (DEFL228/SCRL3)**	*AT1G08695*	10/86	0.887	39.072 ***	11.2% (ω = 2.687)	10N, 17L, 20M, 22T, 24K, 35A, 47N, 49R, 50F, 54L, 56Q, 59T, 61A, 62T, 63Q, 64I, 65S, 67S, 71Q, 82R
**A8MR88 (PCP-Bγ/ESFL8)**	*AT2G16535*	9/72	1.061	34.833 ***	26.3% (ω = 2.147)	5S, 6V, 7E, 9S, 11A, 17T, 18L, 21S, 22V, 23N, 25D, 26N, 27K, 29D, 30R, 31N, 32L, 33T, 38L, 41A, 42K, 43S, 44K, 46R, 49S, 52S, 55V, 57D, 59K, 60D
**A8MQY8 (PCP-Bδ/ESFL9)**	*AT2G16505*	10/85	1.357	74.217 ***	22.0% (ω = 2.975)	14E, 18K, 19S, 20V, 21E, 22S, 23N, 24K, 25A, 26M, 28P, 29V, 31M, 32P, 33V, 34N, 37N, 38N, 39K, 40D, 43K, 44K, 45L, 46T, 48A, 51I, 52G, 53A, 54N, 56P, 57R, 58N, 59R, 62N, 63S, 64R, 65S, 66Q, 68T, 69A, 70D, 73L
**Q9C9N7 (LTP2.5)**	*AT1G66850*	10/81	0.230	2.047	0.5% (ω = 3.321)	NA
**Q2V3C1 (LTP1.9)**	*AT4G33355*	10/97	0.405	5.103	5.8% (ω = 1.876)	NA
**Q8LFM2 (GASA10)**	*AT5G59845*	10/64	0.196	0.160	16.1% (ω = 1.015)	NA

## Data Availability

The datasets generated from this study are available from the corresponding authors upon reasonable request. Mass spectrometry raw data files are available in the public database at ProteomeXchange (https://massive.ucsd.edu) with the project ID MSV000090853.

## Supplementary Materials

The following are available online at https://www.mdpi.com/article/10.3390/biom13010157/s1, Figure S1: Relative abundance of the proteins identified from each proteomic dataset of the pollen coat from *Arabidopsis thaliana,* Figure S2: Gene ontology (GO) enrichment of the *Arabidopsis thaliana* pollen coat proteome, Figure S3: Gene ontology (GO) enrichment of putative orthologs in *Arabidopsis thaliana* of the *Arabidopsis lyrata* pollen coat proteome, Figure S4: Gene ontology (GO) enrichment of putative orthologs in *Arabidopsis thaliana* of the *Brassica oleracea* pollen coat proteome, Table S1: Peptides detected from the *Arabidopsis thaliana* pollen coat, Table S2: Peptides detected from the *Arabidopsis lyrata* pollen coat, Table S3: Peptides detected from the *Brassica oleracea* pollen coat, Table S4: Proteins detected from the *Arabidopsis thaliana* pollen coat, Table S5: Proteins detected from the *Arabidopsis lyrata* pollen coat, Table S6: Proteins detected from the *Brassica oleracea* pollen coat, Table S7: Gene ontology (GO) enrichment analyses of the *Arabidopsis thaliana* pollen coat proteome, Table S8: Gene ontology (GO) enrichment analyses of the *Arabidopsis lyrata* pollen coat proteome, Table S9: Gene ontology (GO) enrichment analyses of the *Brassica oleracea* pollen coat proteome, Table S10: Categories of proteins identified in the pollen coat from *Arabidopsis thaliana*, *Arabidopsis lyrata*, and *Brassica oleracea*, Table S11: Cysteine-rich proteins (CRPs) detected from the *Arabidopsis thaliana* pollen coat, Table S12: Cysteine-rich proteins (CRPs) detected from the *Arabidopsis lyrata* pollen coat, Table S13: Cysteine-rich proteins (CRPs) detected from the *Brassica oleracea* pollen coat, Table S14: Lists of *Arabidopsis thaliana* pollen coat proteins or constituent genes that overlap with previously reported proteomes and transcriptomes.
