# Supplementary material for: Pollen Coat Proteomes of Arabidopsis thaliana, Arabidopsis lyrata, and Brassica oleracea Reveal Remarkable Diversity of Small Cysteine-Rich Proteins at the Pollen-Stigma Interface
Source: Biomolecules. 2023 Jan 12;13(1):157. doi: 10.3390/biom13010157 (PMC9856046; doi:10.3390/biom13010157)
Supplement: Supplementary file 1 [file biomolecules-13-00157-s001.zip › Supplemental materials Figure S1-S4.pdf]

Supplemental Materials

Article title: Pollen coat proteomes of *Arabidopsis thaliana*, *Arabidopsis lyrata* and *Brassica oleracea* reveal remarkable diversity of small cysteine-rich proteins at the pollen-stigma interface

Authors: Ludi Wang, Yui Leung Lau, Fan Lian, Maurice Bosch and James Doughty

The following Supplemental Materials are available for this article:

Figure S1. Relative abundance of the proteins identified from each proteomic dataset of the pollen coat from *Arabidopsis thaliana.*

Figure S2. Gene ontology (GO) enrichment of the *Arabidopsis thaliana* pollen coat proteome.

Figure S3. Gene ontology (GO) enrichment of putative orthologs in *Arabidopsis thaliana* of the *Arabidopsis lyrata* pollen coat proteome.

Figure S4. Gene ontology (GO) enrichment of putative orthologs in *Arabidopsis thaliana* of the *Brassica oleracea* pollen coat proteome.

Table S1. Peptides detected from the *Arabidopsis thaliana* pollen coat.

Table S2. Peptides detected from the *Arabidopsis lyrata* pollen coat.

Table S3. Peptides detected from the *Brassica oleracea* pollen coat.

Table S4. Proteins detected from the *Arabidopsis thaliana* pollen coat.

Table S5. Proteins detected from the *Arabidopsis lyrata* pollen coat.

Table S6. Proteins detected from the *Brassica oleracea* pollen coat.

Table S7. Gene ontology (GO) enrichment analyses of the *Arabidopsis thaliana* pollen coat proteome.

Table S8. Gene ontology (GO) enrichment analyses of the *Arabidopsis lyrata* pollen coat proteome.

Table S9. Gene ontology (GO) enrichment analyses of the *Brassica oleracea* pollen coat proteome.

Table S10. Categories of proteins identified in the pollen coat from *Arabidopsis thaliana*, *Arabidopsis lyrata* and *Brassica oleracea*.

Table S11. Cysteine-rich proteins (CRPs) detected from the *Arabidopsis thaliana* pollen coat.

Table S12. Cysteine-rich proteins (CRPs) detected from the *Arabidopsis lyrata* pollen coat.

Table S13. Cysteine-rich proteins (CRPs) detected from the *Brassica oleracea* pollen coat.

Table S14. Lists of *Arabidopsis thaliana* pollen coat proteins or constituent genes that overlap with previously reported proteomes and transcriptomes.

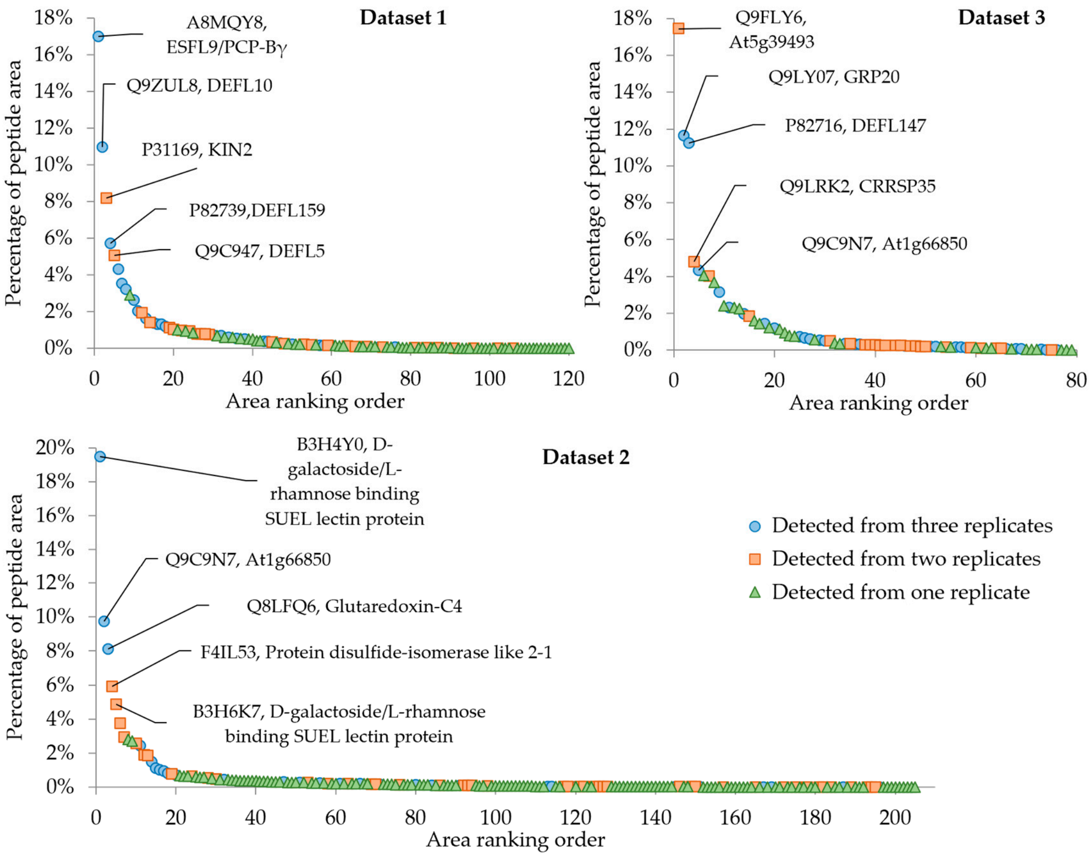

Figure S1. Relative abundance of the proteins identified from each proteomic dataset of the pollen coat from *Arabidopsis thaliana*. Relative abundance was estimated based on the percentage of peptide area in each dataset (Table S4).

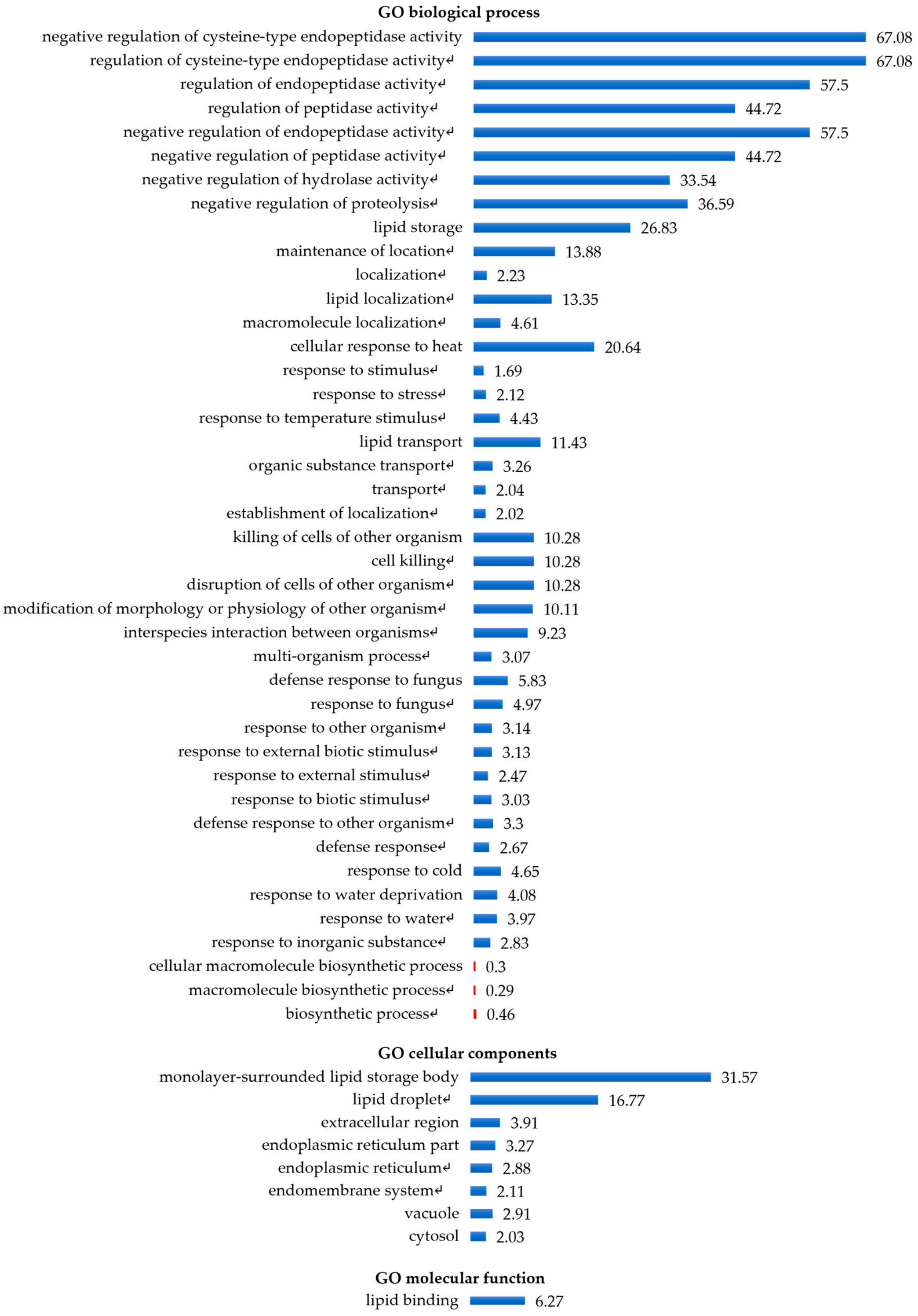

Figure S2. Gene ontology (GO) enrichment of the *Arabidopsis thaliana* pollen coat proteome. The arrows indicate the hierarchy of the categories: terms of the most specific subclasses are shown without arrow, followed by the parent terms shown below indicated with arrow. Numbers beside bars demonstrate the Fold Enrichment of GO terms over the expected. Fold numbers >1 indicate overrepresented categories. Fold numbers <1 are underrepresented categories. Only the categories with p-values < 0.05 (determined by Fisher's exact test) are shown.

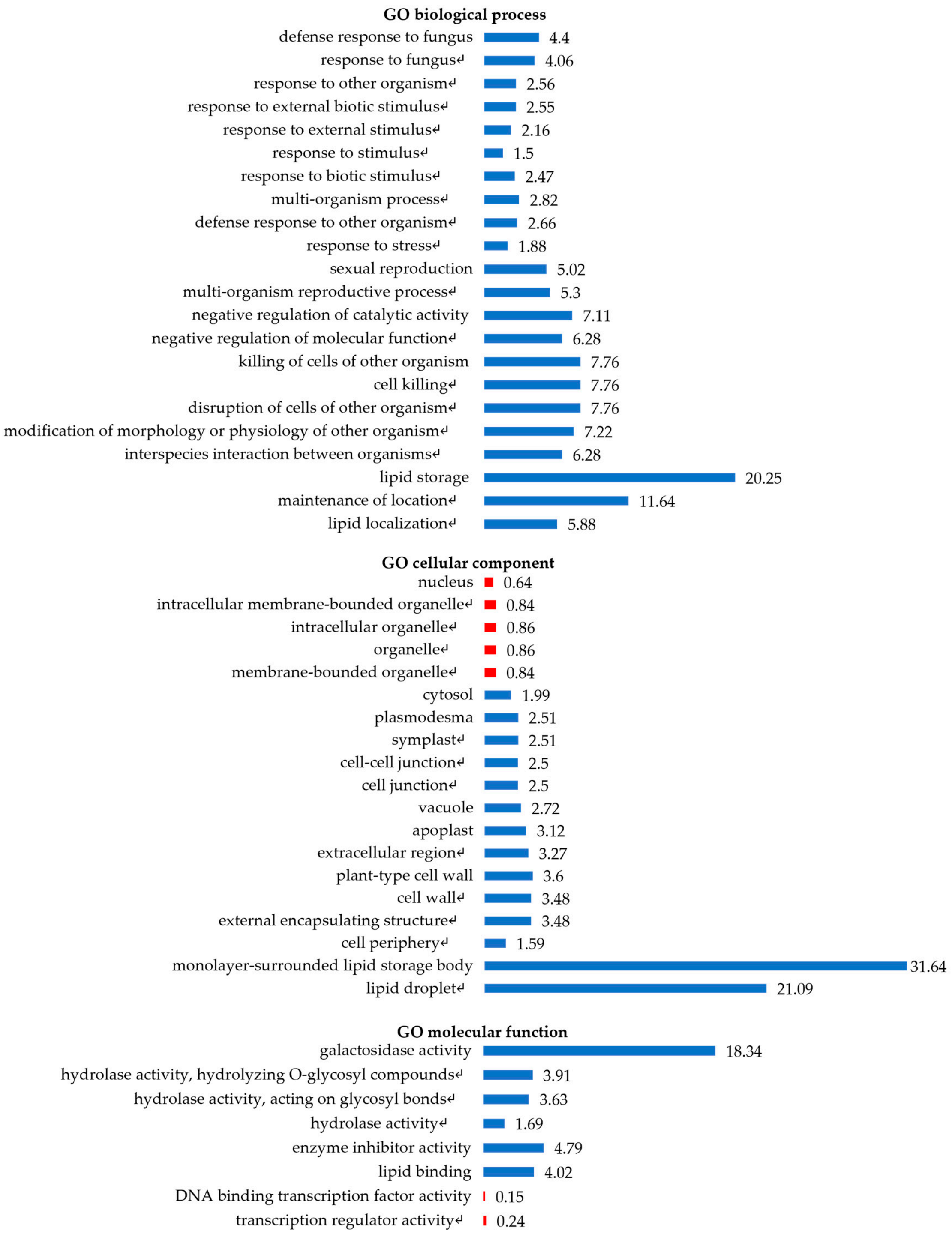

Figure S3. Gene ontology (GO) enrichment of putative orthologs in *Arabidopsis thaliana* of the *Arabidopsis lyrata* pollen coat proteome. The arrows indicate the hierarchy of the categories: terms of the most specific subclasses are shown without arrow, followed by the parent terms shown below indicated with arrow. Numbers beside bars demonstrate the Fold Enrichment of GO terms over the expected. Fold numbers > 1 indicate overrepresented categories. Fold numbers < 1 are underrepresented categories. Only the categories with p-values < 0.05 (determined by Fisher's exact test) are shown.

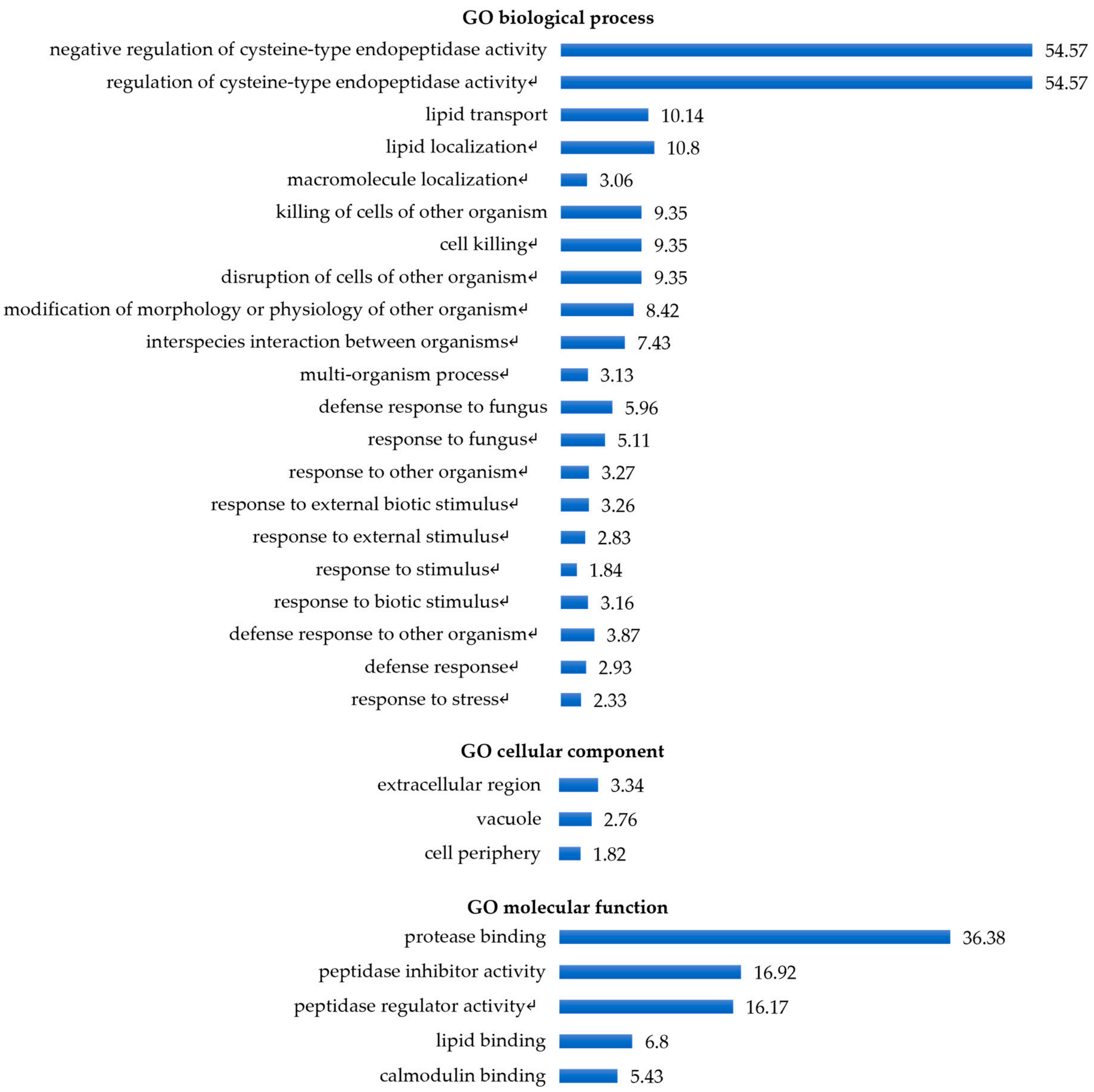

Figure S4. Gene ontology (GO) enrichment of putative orthologs in *Arabidopsis thaliana* of the *Brassica oleracea* pollen coat proteome. The arrows indicate the hierarchy of the categories: terms of the most specific subclasses are shown without arrow, followed by the parent terms shown below indicated with arrow. Numbers beside bars demonstrate the Fold Enrichment of GO terms over the expected. Fold numbers > 1 indicate overrepresented categories. Only the categories with *p*-values < 0.05 (determined by Fisher's exact test) are shown.
